# Crystallographic Effects on Residual Relative Elastic Strain Heterogeneity Induced by Micro-Indentation in Non-Oriented Electrical Steels

**DOI:** 10.3390/ma19102056

**Published:** 2026-05-14

**Authors:** Oluwasogo Adegboyega, Nicolas Brodusch, Lise Guichaoua, Stéphanie Bessette, Richard R. Chromik, Raynald Gauvin

**Affiliations:** Department of Mining and Materials Engineering, McGill University, M.H. Wong Building, 3610 University Street, Montreal, QC H3A 0C5, Canada; oluwasogo.adegboyega@mail.mcgill.ca (O.A.);

**Keywords:** HR-EBSD, micro-indentation, elastic strain, crystallographic orientation

## Abstract

Localized mechanical loading induces complex elastic–plastic interactions in anisotropic crystalline materials. However, quantitative orientation-resolved characterization of residual relative elastic strain heterogeneity remains limited. In this study, high-resolution electron backscatter diffraction was used to map residual in-plane relative elastic strain distributions beneath micro-indents in two annealed body-centered cubic ferritic non-oriented electrical steels, B35AV1900 and 35WW300. Grains oriented near (001), (101), and (111) were analyzed to evaluate the crystallographic effects on residual strain accommodation. Frequency distributions of the in-plane residual relative elastic strain components were constructed, and full width at half maximum values were extracted to quantify strain heterogeneity. The results revealed a pronounced orientation dependence. Near-(001) grains exhibited greater indentation depths and more widely distributed post-indentation deformation features. By contrast, near-(111) grains showed broader residual in-plane relative elastic strain distributions in both alloys. These results indicate that residual strain heterogeneity after unloading is influenced not only by indentation depth but also by crystallographic constraint and orientation-dependent strain redistribution. This study establishes a quantitative orientation-resolved framework for characterizing residual relative elastic strain heterogeneity beneath localized loading. It also provides a basis for linking crystallographic anisotropy, localized deformation, and residual strain redistribution in ferritic electrical steels.

## 1. Introduction

Elastic strain distributions and plastic deformation are strongly influenced by crystallographic orientation, slip system activation, and local constraints [1,2,3,4]. As plastic deformation governs permanent shape changes and dislocation accumulation, elastic strain fields play an important role in stress redistribution, elastic compatibility between neighboring regions, and residual mechanical state after unloading. These residual elastic strains are especially important under localized loading. They reflect elastic back-stresses caused by spatially non-uniform plastic deformation and reveal how microstructure and crystallography influence deformation beyond the immediate plastic zone [5,6,7,8].

Therefore, experimental techniques capable of resolving elastic strain fields at high spatial resolution are necessary to directly quantify residual stress and strain heterogeneity associated with indentation-induced deformation.

Conventional electron backscatter diffraction (EBSD) is widely used to characterize deformation using lattice orientation mapping and misorientation-based metrics [5,9,10]. Curvature-based methods derived from conventional EBSD also enable qualitative assessment of geometrically necessary dislocation (GND) density and plastic strain localization. However, the angular resolution of this indexing-based technique is fundamentally limited. This reduces its sensitivity to measure small elastic lattice strains and makes it ineffective for measuring elastic strain below ≈10^−3^ [5,7,11]. In addition, conventional EBSD only provides indirect or qualitative information regarding elastic strain fields associated with localized deformation.

The development of cross-correlation-based high-resolution EBSD (HR-EBSD) marked a major advancement in microscale strain measurement [3,12]. By correlating electron backscatter diffraction patterns (EBSPs) rather than relying solely on band indexing, HR-EBSD enables quantitative assessment of elastic strain and lattice rotation with sensitivities approaching 10^−4^ or better [8,12]. This capability led to the development of robust methodologies for extracting in-plane elastic strain tensors and showed that HR-EBSD can resolve subtle elastic strain variations that conventional EBSD cannot detect.

HR-EBSD has since been applied to a wide range of deformation problems involving elastic strain. For example, Britton et al. used HR-EBSD to map the elastic strain heterogeneity at slip bands and grain boundaries, showing a strong influence of crystallographic orientation and local constraints on elastic strain heterogeneity [7,13]. HR-EBSD has also been used to quantify elastic strain and stress fields near crack tips and interfaces, thereby supporting continuum fracture mechanics predictions with experimentally observed strain distributions [3,5,14]. In crystalline materials, the technique has been widely used to characterize elastic strain fields around hydrides, grain boundaries, and irradiation-induced defects [11,15,16,17]. This technique reveals significant local stress concentrations and elastic–plastic interactions that influence fracture and damage evolution.

Indentation provides a highly controlled framework for HR-EBSD because it produces steep strain gradients within the small volume of the material [1]. HR-EBSD indentation studies have shown that residual relative elastic strain fields remain outside the plastically deformed indent core and are characterized by alternating tensile and compressive regions [5]. By combining nanoindentation with conventional EBSD and HR-EBSD techniques, Britton et al. showed that orientation-dependent anisotropy leads to systematic variation in lattice rotations, elastic strain distributions, and slip activity due to indentation-induced localized deformation [13]. These approaches were later extended to scratches and more complex contact geometries, confirming that elastic strain accommodation under localized loading is fundamentally linked to crystallographic orientation and slip geometry [18]. Prior experimental and simulation studies have also shown that crystal orientation strongly influences indentation-induced residual stress fields and local deformation morphology through plastic anisotropy and crystallography-dependent slip activation [13,19,20].

Despite these advances, most HR-EBSD indentation studies still rely on spatial strain maps and localized extremes to describe residual relative elastic strain fields. Although such maps provide qualitative insights into deformation morphology, quantitative comparisons are often influenced by spatially variable pattern quality, varying measurement precision, and isolated high-gradient regions [5,7,15]. This is particularly evident within or near the indentation core, where severe lattice distortion can degrade EBSP quality and increase measurement uncertainty. As a result, statistical descriptors derived from the full strain-field distribution may provide a more robust basis for comparing orientations, materials, and processing conditions, with reduced sensitivity to local outliers.

Beyond recent advances in elastic strain measurements, experimental evidence also shows that localized deformation strongly affects the microstructural and mechanical heterogeneity in non-oriented electrical steels (NOES), particularly during secondary manufacturing processes [21,22]. Processes such as cutting, punching, coating, indentation, interlocking, and joining, can introduce plastic deformation, hardness gradients, and residual stresses near the surface [23,24,25], which in turn influence NOES performance and magnetic losses [26,27].

Although previous studies established the presence and spatial extent of processing-induced plastic deformation and residual stress in NOES, the mechanical descriptors used, such as hardness, pop-in behaviors, conventional EBSD, and qualitative microstructural observations, only provide indirect insight into the associated elastic strain fields. In addition, conventional EBSD-based curvature analysis lacks the angular sensitivity required to detect small elastic strains or subtle orientation-dependent differences [5]. Statistical descriptors for quantifying elastic strain heterogeneity under localized deformation remain underused, even within the broader HR-EBSD literature. Frequency distributions of strain components provide a more robust basis for comparing deformation fields than isolated extremes. This approach is also supported by previous studies showing that elastic strain variability is strongly influenced by microstructural constraint and lattice rotation [5,7]. Histogram-based metrics such as full width at half maximum (FWHM) have been applied to HR-EBSD strain data in irradiation studies [17,28], but their systematic application to indentation-induced, orientation-dependent elastic fields remains limited.

Hence, this study combines micro-indentation with HR-EBSD strain mapping and distribution-based descriptors, such as frequency histograms and FWHM, to quantify orientation-dependent residual relative elastic strain heterogeneity in NOES. Residual strain distributions for grains oriented near (001), (101) and (111) provide a reproducible basis for comparison that is less sensitive to isolated extrema than spatial maps alone. By correlating these descriptors with indentation plastic work and microstructural context, this study provides a quantitative evaluation of crystallography-dependent elastic strain accommodation under localized loading.

## 2. Materials and Experimental Techniques

### 2.1. Materials and Methods

Two commercial NOES grades, B35AV1900 and 35WW300, each with a thickness of approximately 0.35 mm, were examined. These alloys were selected as representative industrial NOES commonly used in motor laminations and electric machine cores [29,30,31]. Their selection enables comparison of orientation-dependent residual relative elastic strain responses across two commercially relevant ferritic BCC NOES grades with modest compositional differences [32,33]. The nominal chemical compositions are provided in Table 1. Before micro-indentation and HR-EBSD analyses, the sheets were annealed at 700 °C for 2 h and furnace-cooled to reduce residual stresses from processing and to promote microstructural stability [21]. Samples were sectioned from the sheets, mounted in conductive epoxy, and prepared through sequential grinding with SiC papers up to 1200 grit, diamond polishing to 0.5 µm, and vibratory polishing in 0.05 µm colloidal silica for approximately 24 h to minimize surface deformation and achieve high-quality EBSD patterns suitable for HR-EBSD analyses.

Table 2 shows the nominal average grain sizes and crystallographic texture determined by EBSD for the two alloys. Saleem et al. reported that lower-loss NOES grades exhibit coarser grain structures, while higher-loss grades have finer average grain sizes. The variations in the grain size influence the magnetic domain wall motion and the localization of plastic deformation during secondary manufacturing processes such as punching and cutting [23].

### 2.2. Instrumented Micro-Indentation Testing

Micro-indentation was performed with an Anton Paar instrument (Graz, Austria) equipped with a Vickers diamond indenter under load control. The instrument was calibrated before testing according to the manufacturer’s procedure, using fused quartz as the reference material. Indentations were performed at a maximum load of 250 mN, with loading and unloading rates of 1500 mN/min and a 10 s hold at peak load. Indents were placed at the center of pre-selected grains and at a sufficient distance from visible grain boundaries to minimize grain boundary effects. Grain orientations were determined by EBSD before indentation, and grains oriented near (001), (101), and (111) were selected for subsequent HR-EBSD strain mapping. Multiple indents were performed for each nominal orientation. Specifically, three indents were conducted for near-(101) and near-(111) grains, while only two indents were conducted for near-(001) grains due to the relative scarcity of suitably isolated near-(001) grains in the analyzed microstructure.

### 2.3. HR-EBSD Analyses

**Figure 1:**
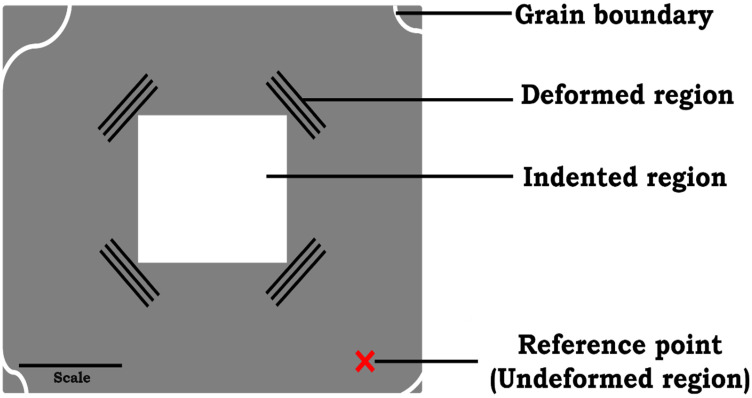
Schematic illustration of the selection of reference pattern for HR-EBSD analysis. The reference point is selected within the same grain, specifically in an undeformed region distant from both the indentation-affected zone and grain boundaries. Pixels that do not meet the correlation quality criterion (MAE > 0.004 rad) are excluded.

High-resolution EBSD measurements were performed using a field-emission SEM (SU-8230, Hitachi High technologies, Inc., Toronto, ON, Canada) equipped with a Bruker eFlash EBSD detector and an ARGUS forescatter detector (Bruker Nano GmbH, Berlin, Germany) for grain-contrast imaging. Patterns were collected at 20 kV, a 26 mm working distance, and a 160 nm step size with zero gain. Cross-correlation HR-EBSD was performed with Crosscourt (v4.6, BLG Vantage) to extract in-plane elastic strain components and lattice rotations from Kikuchi patterns [35]. This method typically achieves a strain sensitivity on the order of 10^−4^ and a rotation sensitivity on the order of 10^−4^ radians under optimal pattern-quality conditions [36,37,38,39]. In this study, the reported strain-distribution widths reflect contributions from both physical residual strain variation and finite measurement broadening. Therefore, the FWHM values are interpreted in a comparative manner across different orientations and alloys, rather than as deconvolved widths. All reported elastic strains are residual relative elastic strains (after unloading), measured with respect to the selected grain reference pattern. They therefore represent elastic strain differences relative to the selected reference point, rather than absolute strain values. A schematic of how the reference pattern was selected (marked in red) across the three distinct orientations is illustrated in Figure 1. For each grain, the reference pattern was chosen from an undeformed region within the same grain, away from the indentation-affected zone and grain boundaries, to minimize strain gradients and maximize pattern fidelity [16,28,35,40]. These analyses focus on the residual in-plane elastic strain components, $\varepsilon_{11}^{e}$, $\varepsilon_{22}^{e}$, and $\varepsilon_{12}^{e}$, measured near the free surface. While HR-EBSD can be used to estimate the elastic strain tensor, the interpretation of the out-of-plane component, $\varepsilon_{33}^{e}$, and the full hydrostatic strain contribution is more sensitive to the traction-free or plane-stress assumption and free-surface relaxation. Accordingly, the results are presented here as orientation-dependent variations in the near-surface residual in-plane elastic strain field after unloading, rather than as a comprehensive 3D strain-state reconstruction [28,41,42].

To ensure robust cross-correlation, pixels were filtered using pattern-quality and correlation metrics. Data points with geometric mean pattern height (PH) < 0.3 and mean angular error (MAE) > 0.004 rad were excluded, which is consistent with the established HR-EBSD quality criteria [8,16,43,44].

**Figure 2:**
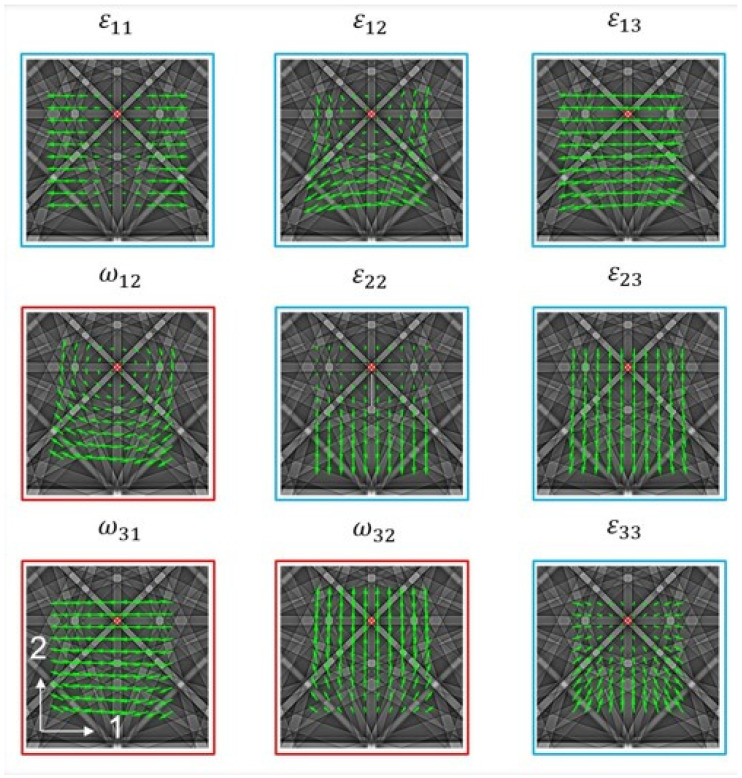
Schematic originally presented by Britton [45], which illustrates the sensitivity of individual deformation-gradient components to EBSD pattern shifts.

Figure 2 summarizes the sensitivity of cross-correlation to individual deformation-gradient components, supporting interpretation of the extracted strain and rotation field distributions. This analysis focuses on the in-plane elastic strain components ($\varepsilon_{11}^{e}$, $\varepsilon_{22}^{e}$, $\varepsilon_{12}^{e}$), which are directly resolved in the surface HR-EBSD configuration and are most relevant to the indentation-induced near-surface strain field.

## 3. Results and Discussions

### 3.1. Microstructure and Grain Orientation Before Micro-Indentation

**Figure 3:**
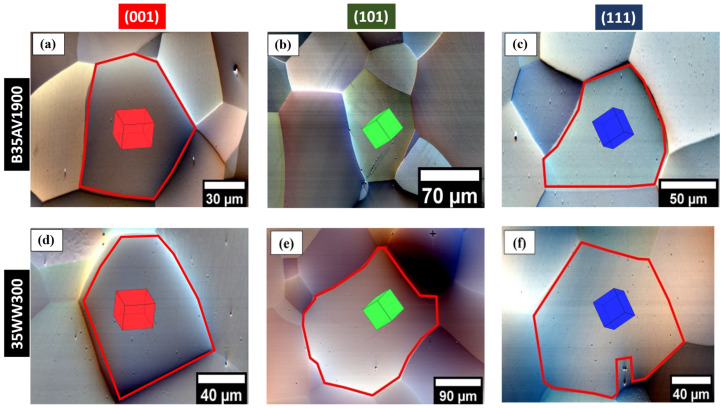
Representative ARGUS forescatter electron (FSE) micrographs of grains in NOES B35AV1900 [(**a**–**c**)] and 35WW300 [(**d**–**f**)] before micro-indentation. The individual grains chosen for analyses are indicated by the red-outlined patches with the unit cells, which correspond to the crystallographic orientations (001), (101), and (111): (**a**,**d**), (**b**,**e**), and (**c**,**f**), respectively. The ARGUS detector provides a baseline for evaluating post-indentation deformation behaviors by detecting magnetic contrast and orientation-dependent channeling inside the grains.

Grain-contrast imaging was performed prior to micro-indentation to document the local microstructure and to identify grains oriented near (001), (101) and (111), as shown in Figure 3 for both NOES grades. The highlighted grains exhibit no visible evidence of pre-existing deformation features at the examined surface scale, supporting their selections as representative nominally stress-relieved regions following annealing. Intragranular intensity variations occasionally observed in the ARGUS contrast are attributed to orientation- and magnetization-sensitive channeling contrast, rather than surface relief, which aligns with previous reports of contrast mechanisms in ferritic steels [46,47]. These baseline micrographs provide the geometric context necessary for subsequent comparisons of indentation morphology and HR-EBSD residual strain fields across different orientations and alloys. Additional pre-indentation ARGUS micrographs for each of the orientations are presented in Figures S1 and S2 of the Supplementary Materials.

### 3.2. Microstructure After Micro-Indentation

**Figure 4:**
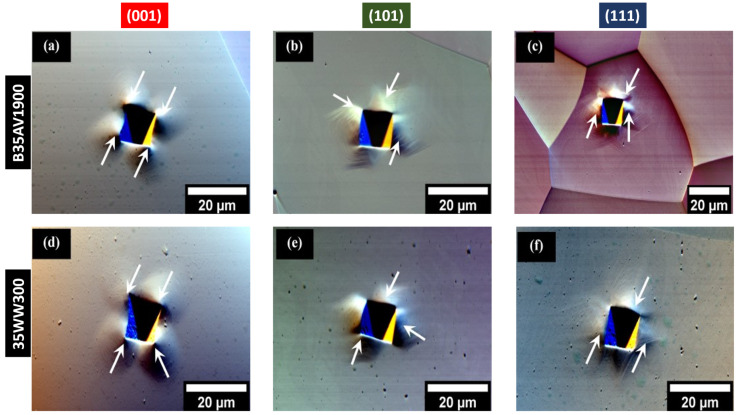
Representative EBSD grain-contrast micrographs of indented grains in NOES B35AV1900 [(**a**–**c**)] and 35WW300 [(**d**–**f**)] following micro-indentation. The crystallographic orientations of the indented grains are (001), (101), and (111): (**a**,**d**), (**b**,**e**), and (**c**,**f**). The white arrows show the strain localization along the crystallographic planes surrounding the indented regions. The two alloys’ microstructural images are compared to show orientation-dependent deformation behaviors and variations in the degree of plastic zone development.

Post-indentation images (Figure 4) show that deformation morphology depends on orientation, with differences in slip traces, plastic zone symmetry, and the extent of surface relief around the indents. Grains near (001) display more distributed deformation, while (111) grains exhibit confined deformation with less lateral spread. The (101) orientation shows intermediate behavior. These qualitative differences demonstrate that crystallographic constraints significantly affect how deformation is distributed beneath the indenter, thereby justifying the subsequent orientation-resolved HR-EBSD analyses.

In both materials, the (001) grains exhibit the highest penetration depths during micro-indentation, followed by (101) and then (111), which demonstrates orientation-dependent resistance to indentation in BCC ferrite. This sequence aligns with crystallographic anisotropy in elastic–plastic accommodation and provides an independent mechanical measure that complements the HR-EBSD strain heterogeneity metrics presented below. The depth values summarized in Table 3 were determined from the complete set of indents for each orientation, including the additional datasets provided in the Supplementary Materials.

To contextualize the observed orientation-dependent deformation behavior, the average Schmid factor values for the selected grains are listed in Table 4. The values reported were also calculated from the complete set of selected grains for each orientation, including the additional datasets provided in the Supplementary Materials. The near-(001) grains show the highest Schmid factors, the near-(101) grains are intermediate, and the near-(111) grains show the lowest values in both alloys. Although the Schmid factor is not by itself sufficient to predict the full deformation response under the complex multiaxial stress state beneath an indenter, this trend is consistent with the more extensive and comparatively symmetric deformation observed around the (001) indents and the more localized, constrained deformation observed for the (111) orientation. These observations suggest easier slip accommodation in near-(001) grains and more restricted deformation in near-(111) grains. The implications of this trend for the broader residual relative elastic strain distributions observed for the near-(111) orientation is discussed later in the paper. Also, a more detailed discussion of these microstructural features, load–displacement curves, modulus, hardness, activated slip systems, plastic work, and geometrically necessary dislocations for these orientations has been presented in our preliminary work [48].

**Figure 5:**
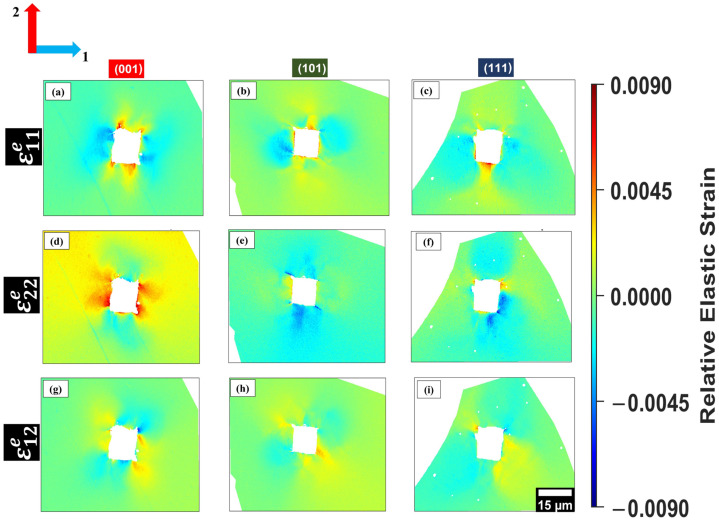
In-plane relative elastic strain maps obtained from HR-EBSD for B35AV1900 grains oriented near (001), (101), and (111): (**a**,**d**,**g**), (**b**,**e**,**h**), and (**c**,**f**,**i**), respectively. The color scale indicates compressive (blue) and tensile (red) regions while the axes 1 and 2 correspond to the horizontal and vertical in-plane directions, respectively, used for strain component calculation. The 15 μm scale bar shown in panel (**i**) applies to all panels.

**Figure 6:**
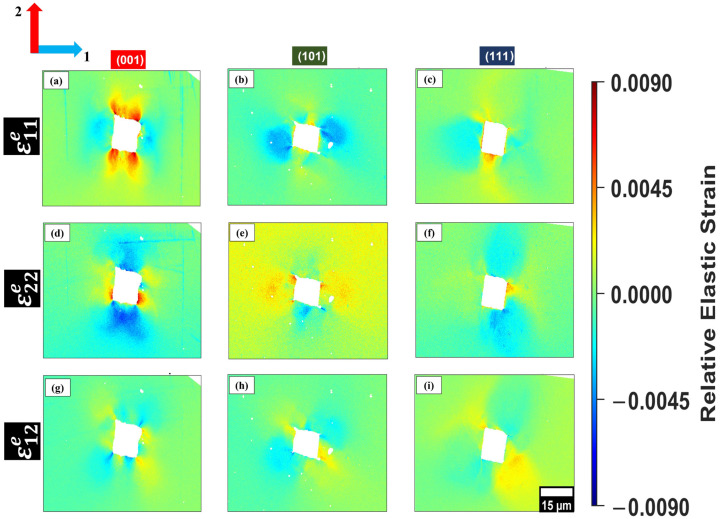
In-plane relative elastic strain maps obtained from HR-EBSD for 35WW300 grains oriented near (001), (101), and (111): (**a**,**d**,**g**), (**b**,**e**,**h**), and (**c**,**f**,**i**), respectively. The color scale indicates compressive (blue) and tensile (red) regions while the axes 1 and 2 correspond to the horizontal and vertical in-plane directions, respectively, used for strain component calculation. The 15 μm scale bar shown in panel (**i**) applies to all panels.

Figure 5 and Figure 6 present the in-plane residual relative elastic strain components ($\varepsilon_{11}^{e}$, $\varepsilon_{22}^{e}$, $\varepsilon_{12}^{e}$) measured by HR-EBSD around indents in grains oriented near (001), (101) and (111) for B35AV1900 and 35WW300, respectively. These maps illustrate the magnitude and spatial distribution of residual strain fields after unloading, allowing direct comparison of orientation-dependent elastic strain accommodation in the two steels.

The $\varepsilon_{11}^{e}$ component captures the residual in-plane normal strain along axis 1, indicating local tensile/compressive strain redistribution around the indent, which is essential in understanding how elastic energy is accommodated [13]. This strain component describes the equilibrium between crystallographic orientation and lattice constraint influencing both the deformation symmetry and initial strain localization [3].

In all orientations, the residual relative elastic strain fields display adjacent tensile and compressive lobes surrounding the indent. This pattern reflects elastic recovery and compatibility requirements imposed by the plastically deformed zone and the constrained surrounding lattice. The spatial arrangement of these strain fields changes with the crystallographic orientation, reflecting anisotropic deformation and orientation-dependent constraints [13].

Although both materials were annealed under identical conditions, slight variations in $\varepsilon_{11}^{e}$ strain magnitudes and distributions are observed. The (001) grain in 35WW300 has a slightly higher tensile strain compared to its counterpart in B35AV1900. The (101) and (111) grains in 35WW300 show larger, more compressive strain distributions especially in regions with low strain gradients [3]. These discrepancies may not be related to annealing, but rather to small differences in grain misorientations (as detected via EBSD), slight differences in indentation depths and chemical composition.

The $\varepsilon_{22}^{e}$ maps measure the in-plane normal residual strain perpendicular to $\varepsilon_{11}^{e}$. Together, $\varepsilon_{11}^{e}$ and $\varepsilon_{22}^{e}$ define the anisotropic in-plane residual strain around the indent, while $\varepsilon_{12}^{e}$ represents the in-plane shear component related to compatibility and local lattice rotation [35].

**Figure 7:**
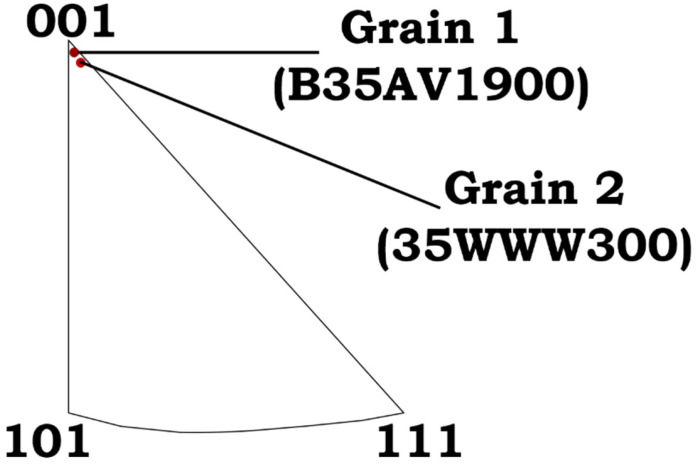
An example of how small variations in selected grain orientations can affect strain gradient distributions in B35AV1900 and 35WW300 NOES.

Both B35AV1900 and 35WW300 exhibit orientation-dependent differences in the $\varepsilon_{22}^{e}$ residual in-plane normal strain field. In grains oriented near (001), B35AV1900 shows a more symmetric arrangement of tensile and compressive $\varepsilon_{22}^{e}$ strain surrounding the indent, while 35WW300 reveals a more spatially diffuse and less symmetric $\varepsilon_{22}^{e}$ distribution. For the (101) orientation, both steels display asymmetric $\varepsilon_{22}^{e}$ fields, but the degree of spatial localization and gradient intensity varies between the materials. In grains oriented near (111), B35AV1900 features more pronounced compressive $\varepsilon_{22}^{e}$ regions at the indent corners, whereas 35WW300 exhibits broader but less intense $\varepsilon_{22}^{e}$ gradients. These observations suggest that crystallographic orientation, in conjunction with local microstructural constraints, governs the spatial redistribution and localization of residual elastic strains beneath indentation. For direct comparison, grains were selected to closely match the nominal (001), (101), and (111) orientations. However, small deviations of approximately 0.7° are expected due to the finite precision of EBSD indexing. Such small offsets may influence the local strain distribution by altering slip accommodation and local constraint, thereby contributing to grain-to-grain variability in strain localization within the same nominal orientation, as shown in Figure 7.

In contrast to $\varepsilon_{11}^{e}$ and $\varepsilon_{22}^{e}$, which describe normal strain along fixed axes, the $\varepsilon_{12}^{e}$ component represents in-plane elastic shear distortion, reflecting shear-dominated deformation and compatibility constraints in the near-surface regions [16,35]. This strain component is especially useful in BCC materials, where shear stresses strongly influence slip initiation and the preferred deformation mode [13]. In addition, Roy (2024) found that $\varepsilon_{12}^{e}$ mapping helps us to understand how strain localizes and relaxes at sub-micron scales under applied stress [16].

In (001) grains, the $\varepsilon_{12}^{e}$ distributions for both B35AV1900 and 35WW300 alloys demonstrate periodic, alternating shear distributions extending diagonally from the indent edges. These symmetric shear distributions indicate that the (001) grains accommodate shear through energetically balanced deformation paths. Britton (2010) and Wilkinson (2010) found that high-symmetry orientations like (001) in BCC metals activate multiple slip systems simultaneously, resulting in more uniformly shear fields with energetically balanced strain accommodation [13,35]. In contrast, (101) grains reveal denser $\varepsilon_{12}^{e}$ regions with directional bias. For B35AV1900, these areas align more closely with a single dominating shear direction, indicating the preferential activation of fewer slip systems. This observation indicates anisotropic shear accommodation, and more localized lattice distortion. In comparison, the (101) grain in 35WW300 has significantly more delocalized shear distribution with lower peak intensity, probably due to the reduced local lattice friction, chemical composition, and shallower indentation depth.

The (111) orientation reveals the most constrained $\varepsilon_{12}^{e}$ distributions in both samples. In these grains, $\varepsilon_{12}^{e}$ is weaker and confined along the indent edges, indicated by the limited lateral shear redistribution and greater resistance to in-plane distortion. These findings agree with Wilkinson et al. (2010) as they observed that the distributions and extent of the $\varepsilon_{12}^{e}$ fields are highly sensitive to crystallographic orientation and local geometrical constraints [35]. Britton (2010) stated that orientation-governed anisotropy in shear deformation becomes especially prominent in areas with high local triaxial stress, such as near the indentation site [13]. Overall, $\varepsilon_{12}^{e}$ strain mapping provides insights into the evolution of in-plane lattice distortion as a function of crystallographic orientation, complementing the normal strain fields by revealing the directional shear anisotropy. The remaining residual relative elastic strain maps for the additional indents are provided in Figures S5–S10 of the Supplementary Materials.

**Figure 8:**
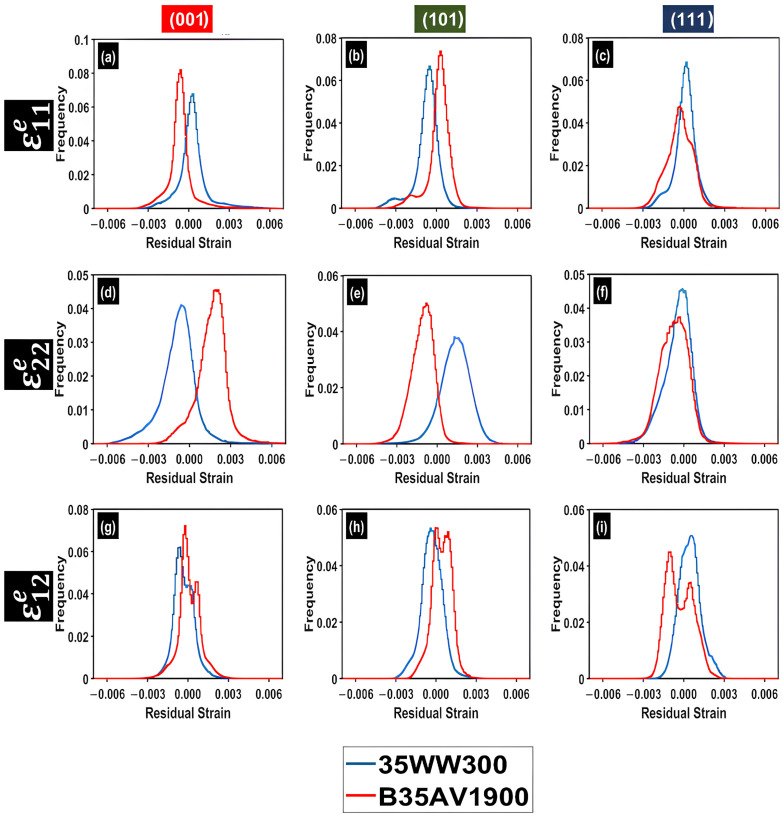
Frequency distributions of the in-plane residual relative elastic strain components for grains oriented near (001), (101) and (111) in B35AV1900 and 35WW300. Subfigures (**a**–**c**) show $\varepsilon_{11}^{e}$, (**d**–**f**) show $\varepsilon_{22}^{e}$, and (**g**–**i**) show $\varepsilon_{12}^{e}$. Within each row, the columns correspond to near-(001), near-(101), and near-(111) grains, respectively. The distributions were constructed from pixels within the elastically deformed region around the indentation, excluding the indentation core, after trimming the data to the 95% confidence interval.

To move beyond pointwise interpretation of spatial maps, residual relative elastic strain fields were quantified using frequency distributions of $\varepsilon_{11}^{e}$, $\varepsilon_{22}^{e}$ and $\varepsilon_{12}^{e}$ sampled from the elastically deformed regions surrounding each indent, as shown in Figure 8. HR-EBSD strain fields were used to construct frequency distributions by sampling pixels within the elastically deformed region surrounding each indentation. However, the indentation cores were excluded due to the severe lattice distortion and poor Kikuchi patterns, which could affect the measurement accuracy if not filtered. The remaining frequency distribution plots for the additional indents are provided in Figure S12 of the Supplementary Materials.

This distribution-based approach captures both the central tendency and variability of strain values, providing a more reproducible basis for comparing crystallographic orientations and alloys than reliance on isolated extrema or visually selected features. Systematic differences in distribution width across (001), (101), and (111) orientations demonstrate that crystallographic orientation influences both the spatial morphology of the strain field and the extent of residual relative elastic strain heterogeneity retained after unloading. The distributions exhibit distinct orientation-dependent shifts in peak position and variations in width, with occasional mild asymmetry or bimodality. Quantitative analysis using FWHM indicates that crystallography determines both the magnitude and heterogeneity of residual relative elastic strain following unloading.

To quantify the distribution width using a single scalar metric, the FWHM values of the histograms for each orientation were obtained and are reported in Table 5 (B35AV1900) and Table 6 (35WW300), respectively. The values reported in these tables were determined from the complete set of indents for each orientation, including the additional datasets provided in the Supplementary Materials. FWHM provides a direct measure of the heterogeneity of the residual relative elastic strain distributions. Higher FWHM values indicate broader residual relative elastic strain distributions and increased residual relative elastic strain heterogeneity within the mapped region, while lower FWHM values reflect a narrower and more spatially uniform residual elastic response.

Although the near-(001) grains exhibited higher indentation depths, the FWHM analyses indicate that the near-(111) grains displayed the broadest residual in-plane relative elastic strain distributions in both alloys. Therefore, FWHM should not be interpreted as a direct measure of the overall magnitude of deformation. Rather, it reflects the breadth of the retained residual relative elastic strain distribution after unloading and, hence, the degree of residual strain heterogeneity within the mapped region. This interpretation is consistent with previous HR-EBSD studies in which FWHM was used to characterize the spatial variation in residual strain fields [17,28]. Such studies showed that broader distributions can be observed even when macroscopic strain is lower, primarily due to deformation incompatibility and dislocation-related effects. Previous experimental and simulation studies have also shown that crystal orientation strongly affects indentation-induced residual stress fields and local deformation morphology through plastic anisotropy and crystallography-dependent slip activation [13,19,20]. For the NOES grades investigated here, this behavior is more plausibly attributed to the combined effects of crystallographic constraint, elastic anisotropy, orientation-dependent slip accommodation, and redistribution of the residual field around the indent, rather than simply to deformation magnitude. This interpretation is further supported by our complementary orientation-resolved micro-indentation study on the NOES alloys [48]. That study showed consistent orientation dependence in indentation modulus, hardness, Schmid factor, plastic work and GND density. In particular, near-(001) grains exhibited the highest Schmid factors, the greatest indentation depths, the lowest hardness and indentation modulus values, greater plastic work, the highest average GND densities, and more spatially distributed deformation around the indent. In contrast, near-(111) grains exhibited the lowest Schmid factors, shallower indentation depths, higher hardness and indentation modulus, lower plastic dissipation, lower GND density, and more localized deformation features, indicating a more constrained deformation response [48]. Overall, these trends indicate that the broader FWHM values measured for near-(111) grains arise from stronger crystallographic constraint, local incompatibility, and wider redistribution of the residual elastic field, rather than from greater overall deformation magnitude.

**Figure 9:**
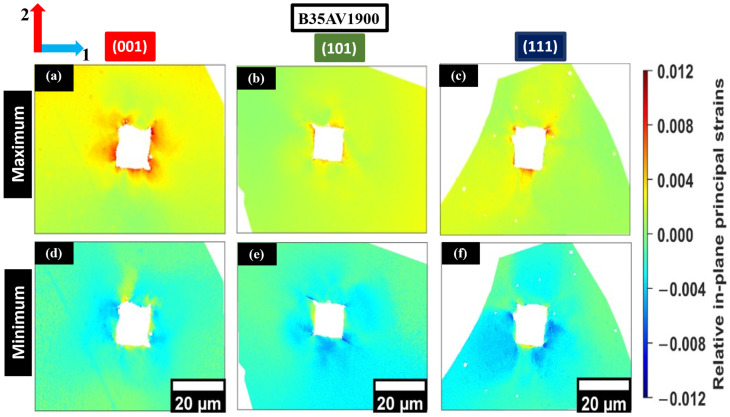
In-plane maximum (**a**–**c**) and minimum (**d**–**f**) relative principal strain maps from HR-EBSD for B35AV1900 grains oriented near (001), (101), and (111). The color scale shows tensile (red) and compressive (blue) regions. Each panel includes its own 20 μm scale bar. Axes 1 and 2 correspond to the horizontal and vertical in-plane directions, respectively.

**Figure 10:**
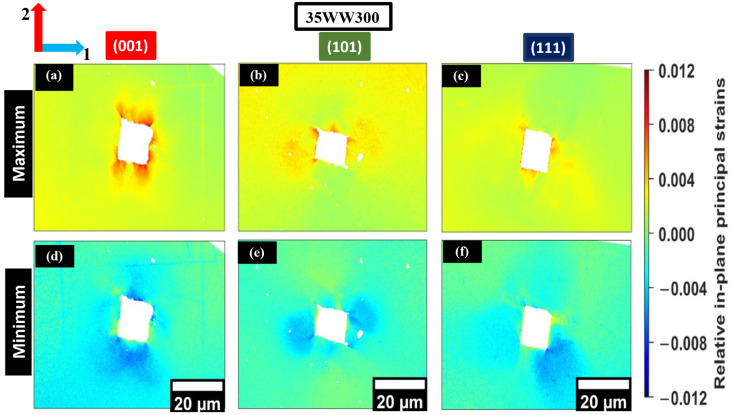
In-plane maximum (**a**–**c**) and minimum (**d**–**f**) relative principal strain maps from HR-EBSD for 35WW300 grains oriented near (001), (101), and (111). The color scale shows tensile (red) and compressive (blue) regions. Each panel includes its own 20 μm scale bar. Axes 1 and 2 correspond to the horizontal and vertical in-plane directions, respectively.

The maximum and minimum relative in-plane principal strains were determined as the eigenvalues of the elastic strain tensor, offering a rotation-invariant view of the local residual relative elastic strain near the indent. Unlike individual tensor components (e.g., $\varepsilon_{11}^{e}$, $\varepsilon_{22}^{e}$ and $\varepsilon_{12}^{e}$), which depend on reference axes, principal strains quantify the largest tensile and compressive normal strains along local principal directions [7,16]. This approach enables clearer comparison of multiaxial elastic distortion across orientations. Figure 9a–c and Figure 10a–c show that the in-plane maximum principal relative elastic strain fields reveal regions where residual elastic tension persists after unloading and how it redistributes around the plastically deformed zone. In both alloys, tensile principal strain is concentrated near the indents; however, its spatial extent and intensity systematically vary with crystallographic orientation, reflecting orientation-dependent constraint and slip accommodation. Variations between nominally similar orientations in the two steels likely result from local microstructural constraints, such as neighboring grain orientations and proximity to grain boundaries, which redistribute elastic strains to maintain intergranular compatibility [13,35].

Orientations associated with deeper penetration and higher plastic accommodation exhibit more localized maximum principal strain patterns. In contrast, orientations with shallower penetration display a more spatially distributed residual elastic field. This trend aligns with variations in indentation depth and the corresponding plastic zone size across orientations. However, local boundary constraints and neighborhood interactions may also influence these observations. The remaining maximum and minimum relative principal strain maps for the additional indents are provided in Figures S13–S16 of the Supplementary Materials.

In contrast, Figure 9d–f and Figure 10d–f show that the in-plane minimum relative principal strain (compressive) field extends over a broader region around the imprints, reflecting the compressive component of the constrained elastic recovery field. Differences in the magnitude and spread of compressive principal strain depend on orientation and align with variations in plastic accommodation. Orientations with more restricted slip activity retain a larger compressive residual field to maintain compatibility with the surrounding lattice [12,13]. Similar broadening of compressive principal strain patterns has been observed in HR-EBSD studies, where local constraint and intergranular interactions govern elastic strain redistribution [5].

## 4. Conclusions

Residual relative elastic strain fields beneath micro-indentation in NOES are strongly influenced by crystallographic orientation, exhibiting systematic differences in strain localization, symmetry, and heterogeneity across grains near (001), (101), and (111). By integrating HR-EBSD strain mapping with distribution-based descriptors such as frequency histograms and FWHM, this study establishes a statistically robust framework for comparing indentation-induced residual relative elastic strain beyond qualitative spatial map inspection. This methodology elucidates how elastic strain redistribution is governed by anisotropic constraint and local microstructural boundary conditions, facilitating clearer, orientation-resolved comparisons between steel grades subjected to identical loading. The findings establish a quantitative pathway for linking crystallography and local deformation mechanics in ferritic steels, and the framework is readily extendable to larger datasets and in situ indentation workflows to further test the generality of strain evolution under different controlled loading conditions.

## Figures and Tables

**Table 1:** Chemical composition of the two annealed NOES grades; B35AV1900 and 35WW300. Both alloys are Fe-Si, with small amounts of Mn, Al, P, S, and C.

Alloy Grade	Chemical Element							Ref.
	Fe (%)	Si (%)	Mn (%)	Al (%)	P (%)	S (%)	C (%)	
B35AV1900	96.100	3.130	0.290	0.440	0.011	0.001	0.011	[34]
35WW300	95.920	3.100	0.260	0.650	0.005	0.002	0.010	

**Table 2:** Nominal average grain size and texture factor of the investigated NOES.

Steel Grade	Grain Size (μm)	Texture Factor	Ref.
35WW300	130 ± 10	0.80	[23,34]
B35AV1900	106 ± 13	0.87	

**Table 3:** Maximum indentation depths obtained in B35AV1900 and 35WW300 NOES for the (001), (101), and (111) crystallographic orientations. Each depth value corresponds to the indentation performed on the specified orientation. All depth values are in μm.

Orientation	(001)	(101)	(111)
B35AV1900	3.24 ± 0.01	3.07 ± 0.04	2.77 ± 0.11
35WW300	2.88 ± 0.02	2.71 ± 0.05	2.58 ± 0.04

**Table 4:** Schmid factor values for selected B35AV1900 and 35WW300 grains oriented near (001), (101) and (111).

Orientation	$\left(001\right)$	$\left(101\right)$	(111)
B35AV1900	0.4970 ± 0.0026	0.4569 ± 0.0102	0.3361 ± 0.0047
35WW300	0.4899 ± 0.0010	0.4677 ± 0.0078	0.3584 ± 0.0091

**Table 5:** FWHM of the in-plane residual relative elastic strain distributions obtained for B35AV1900 grains oriented near (001), (101), and (111) crystallography.

Orientation	$\varepsilon_{11}^{e}$ (×10^−4^)	$\varepsilon_{22}^{e}$ (×10^−4^)	$\varepsilon_{12}^{e}$ (×10^−4^)
(001)	8.19 ± 0.68	14.40 ± 1.05	12.00 ± 1.88
(101)	10.80 ± 1.26	17.40 ± 1.19	15.36 ± 0.43
(111)	13.57 ± 0.39	26.90 ± 0.83	21.30 ± 1.38

**Table 6:** FWHM of the in-plane residual relative elastic strain distributions obtained for 35WW300 grains oriented near (001), (101), and (111) crystallography.

Orientation	$\varepsilon_{11}^{e}$ (×10^−4^)	$\varepsilon_{22}^{e}$ (×10^−4^)	$\varepsilon_{12}^{e}$ (×10^−4^)
(001)	8.07 ± 0.83	15.04 ± 0.68	12.41 ± 0.83
(101)	10.40 ± 1.11	18.50 ± 0.99	14.93 ± 0.65
(111)	13.21 ± 1.46	24.04 ± 1.86	19.65 ± 1.93

## Data Availability

The original contributions presented in the study are included in the article and the Supplementary Materials; further inquiries can be directed to the corresponding authors.

## Supplementary Materials

The following supporting information can be downloaded at: https://www.mdpi.com/article/10.3390/ma19102056/s1, Figure S1: ARGUS forescatter grain-contrast images of grains selected near the (001), (101) and (111) crystallographic orientations in B35AV1900. The overlaid unit cells depict the crystallographic orientation of each selected grain, and their colors follow the inverse pole figure (IPF) colour key used in the EBSD IPF maps.; Figure S2: ARGUS forescatter grain-contrast images of grains selected near the (001), (101) and (111) crystallographic orientations in 35WW300. The overlaid unit cells depict the crystallographic orientation of each selected grain, and their colors follow the inverse pole figure (IPF) colour key used in the EBSD IPF maps.; Figure S3: Grain-contrast (ARGUS forescatter) micrographs of representative Vickers micro-indentations in B35AV1900 grains selected near the (001), (101) and (111) crystallographic orientations. Panels (a–c) shows one representative indent for each orientation, while (d) and (e) show additional indents in grains selected near (101) and (111), respectively.; Figure S4: Grain-contrast (ARGUS forescatter) micrographs of representative Vickers micro-indentations in B35AV1900 grains selected near the (001), (101) and (111) crystallographic orientations. Panels (a–c) shows one representative indent for each orientation, while (d) and (e) show additional indents in grains selected near (101) and (111), respectively.; Figure S5: Additional $\varepsilon_{11}^{e}$ residual in-plane elastic strain maps for extra indents in B35AV1900 grins oriented near (001), (101), and (111). One additional map is shown for near-(001), while two maps are shown for near-(101) and near-(111). All maps are plotted using a common colour scale to facilitate comparison of within-orientation variability and consistency of the observed strain-distribution trends. The 20 μm scale bar shown applies to panels.; Figure S6: Additional $\varepsilon_{22}^{e}$ residual in-plane elastic strain maps for extra indents in B35AV1900 grins oriented near (001), (101), and (111). One additional map is shown for near-(001), while two maps are shown for near-(101) and near-(111). All maps are plotted using a common colour scale to facilitate comparison of within-orientation variability and consistency of the observed strain-distribution trends. The 20 μm scale bar shown applies to panels.; Figure S7: Additional $\varepsilon_{12}^{e}$ residual in-plane elastic strain maps for extra indents in B35AV1900 grins oriented near (001), (101), and (111). One additional map is shown for near-(001), while two maps are shown for near-(101) and near-(111). All maps are plotted using a common colour scale to facilitate comparison of within-orientation variability and consistency of the observed strain-distribution trends. The 20 μm scale bar shown applies to panels.; Figure S8: Additional $\varepsilon_{11}^{e}$ residual in-plane elastic strain maps for extra indents in 35WW300 grins oriented near (001), (101), and (111). One additional map is shown for near-(001), while two maps are shown for near-(101) and near-(111). All maps are plotted using a common colour scale to facilitate comparison of within-orientation variability and consistency of the observed strain-distribution trends. The 20 μm scale bar shown applies to panels.; Figure S9: Additional $\varepsilon_{22}^{e}$ residual in-plane elastic strain maps for extra indents in 35WW300 grins oriented near (001), (101), and (111). One additional map is shown for near-(001), while two maps are shown for near-(101) and near-(111). All maps are plotted using a common colour scale to facilitate comparison of within-orientation variability and consistency of the observed strain-distribution trends. The 20 μm scale bar shown applies to panels.; Figure S10: Additional $\varepsilon_{12}^{e}$ residual in-plane elastic strain maps for extra indents in 35WW300 grains oriented near (001), (101), and (111). One additional map is shown for near-(001), while two maps are shown for near-(101) and near-(111). All maps are plotted using a common colour scale to facilitate comparison of within-orientation variability and consistency of the observed strain-distribution trends. The 20 μm scale bar shown applies to panels.; Figure S11: Frequency distributions of the residual in-plane elastic strain components $\varepsilon_{11}^{e}$, $\varepsilon_{22}^{e}$ and $\varepsilon_{12}^{e}$ are presented for additional indents in B35AV1900 grains oriented near (001), (101) and (111). For the near-(001) grain, one additional indent is displayed. For the near-(101) and near-(111) grains, the blue and red curves represent to the second and third indents, respectively. These plots demonstrate the variability within each orientation and the consistency of strain-distribution trends across repeated indents.; Figure S12: Frequency distributions of the residual in-plane elastic strain components $\varepsilon_{11}^{e}$, $\varepsilon_{22}^{e}$ and $\varepsilon_{12}^{e}$ are presented for additional indents in 35WW300 grains oriented near (001), (101) and (111). For the near-(001) grain, one additional indent is displayed. For the near-(101) and near-(111) grains, the blue and red curves represent to the second and third indents, respectively. These plots demonstrate the variability within each orientation and the consistency of strain-distribution trends across repeated indents.; Figure S13: Additional in-plane relative maximum principal strain maps are provided for extra indents in B35AV1900 grains oriented near (001), (101) and (111). One additional map is shown for near-(001), while two maps are shown for near-(101) and near-(111). All maps are plotted using a common colour scale to facilitate comparison of within-orientation variability and consistency in strain-distribution trends. The 20 μm scale bar applies to all panels.; Figure S14: Additional in-plane relative minimum principal strain maps are provided for extra indents in B35AV1900 grains oriented near (001), (101) and (111). One additional map is shown for near-(001), while two maps are shown for near-(101) and near-(111). All maps are plotted using a common colour scale to facilitate comparison of within-orientation variability and consistency in strain-distribution trends. The 20 μm scale bar applies to all panels.; Figure S15: Additional in-plane relative maximum principal strain maps are provided for extra indents in 35WW300 grains oriented near (001), (101) and (111). One additional map is shown for near-(001), while two maps are shown for near-(101) and near-(111). All maps are plotted using a common colour scale to facilitate comparison of within-orientation variability and consistency in strain-distribution trends. The 20 μm scale bar applies to all panels.; Figure S16: Additional in-plane relative minimum principal strain maps are provided for extra indents in 35WW300 grains oriented near (001), (101) and (111). One additional map is shown for near-(001), while two maps are shown for near-(101) and near-(111). All maps are plotted using a common colour scale to facilitate comparison of within-orientation variability and consistency in strain-distribution trends. The 20 μm scale bar applies to all panels.

## Abbreviations

The following abbreviations are used in this manuscript:

HR-EBSD	High-resolution electron backscatter diffraction
EBSP	Electron backscatter diffraction pattern
NOES	Non-oriented electrical steel
FWHM	Full width at half maximum
GND	Geometrically necessary dislocation
BCC	Body-centered cubic
