# Supplementary material for: Crystallographic Effects on Residual Relative Elastic Strain Heterogeneity Induced by Micro-Indentation in Non-Oriented Electrical Steels
Source: Materials (Basel). 2026 May 14;19(10):2056. doi: 10.3390/ma19102056 (PMC13208754; doi:10.3390/ma19102056)
Supplement: Supplementary file 1 [file materials-19-02056-s001.zip › materials-4234489-supplementary.pdf]

Crystallographic Effects on Residual Relative Elastic Strain Heterogeneity Induced by Micro-Indentation in Non-Oriented Electrical Steels

Oluwasogo Adegboyega, Nicolas Brodusch, Lise Guichaoua, Stéphanie Bessette, Richard R. Chromik * and Raynald Gauvin *

Department of Mining and Materials Engineering, McGill University, M.H. Wong Building, 3610 University Street, Montreal, QC H3A 0C5, Canada

* Correspondence: richard.chromik@mcgill.ca (R.R.C.); raynald.gauvin@mcgill.ca (R.G.)

Supplementary Data

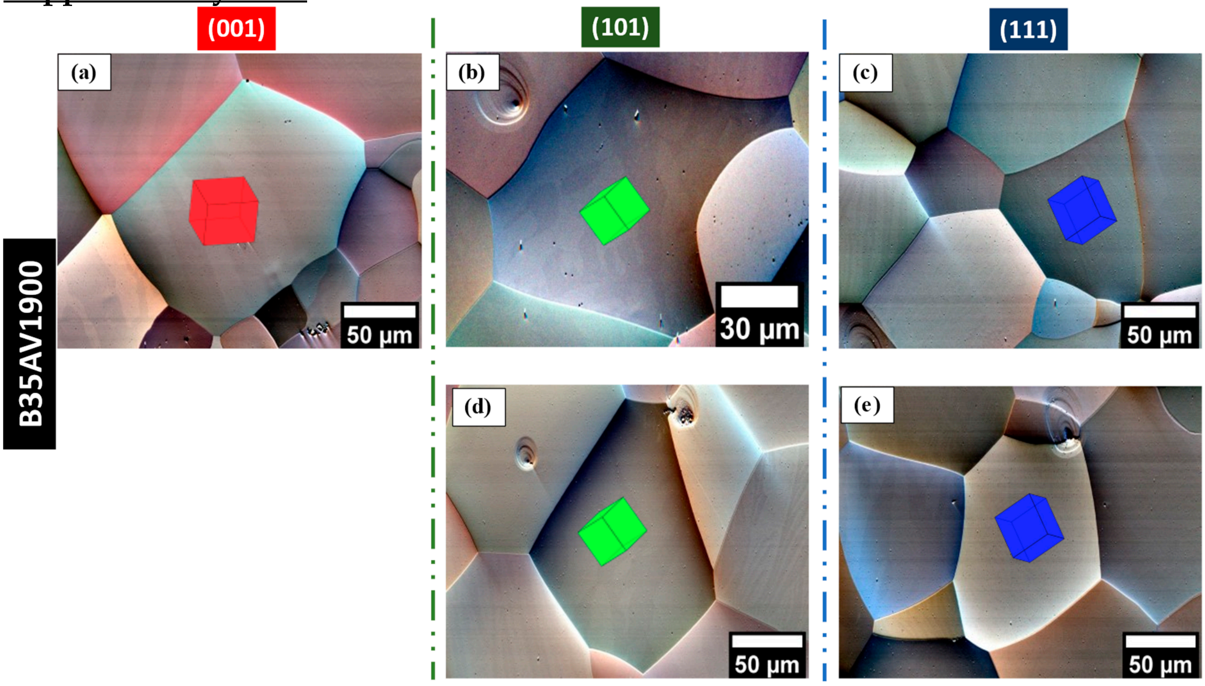

Figure S1 ARGUS forescatter grain-contrast images of grains selected near the (001), (101) and (111) crystallographic orientations in B35AV1900. The overlaid unit cells depict the crystallographic orientation of each selected grain, and their colors follow the inverse pole figure (IPF) colour key used in the EBSD IPF maps.

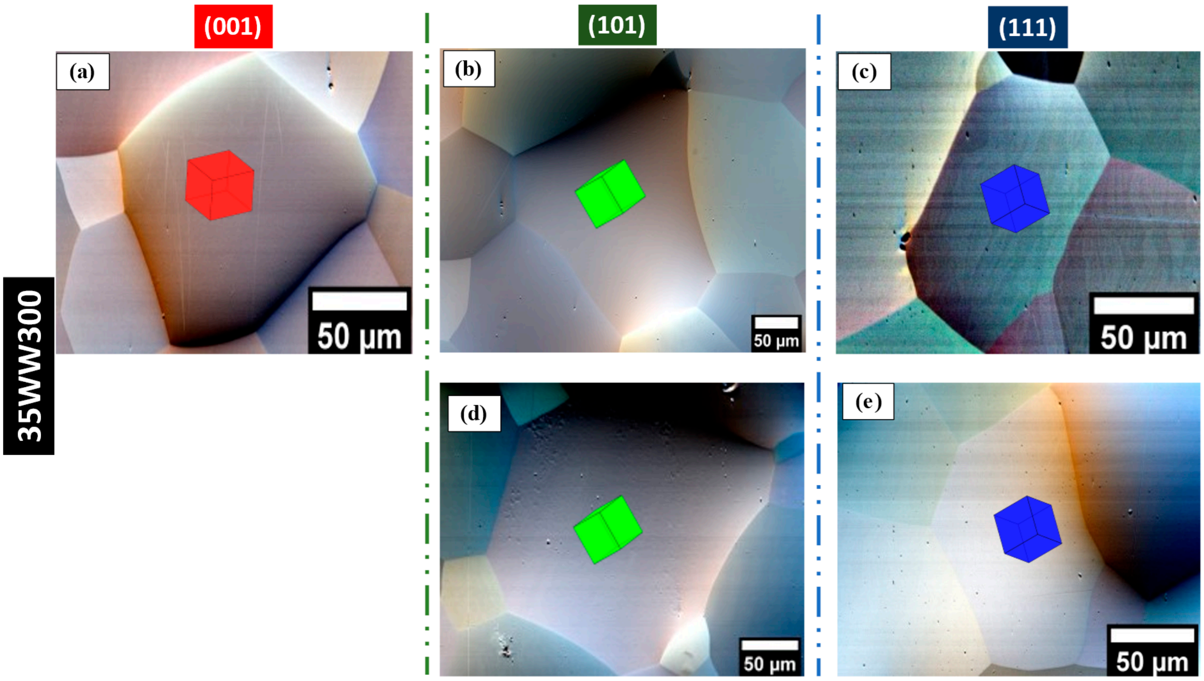

Figure S2 ARGUS forescatter grain-contrast images of grains selected near the (001), (101) and (111) crystallographic orientations in 35WW300. The overlaid unit cells depict the crystallographic orientation of each selected grain, and their colors follow the inverse pole figure (IPF) colour key used in the EBSD IPF maps.

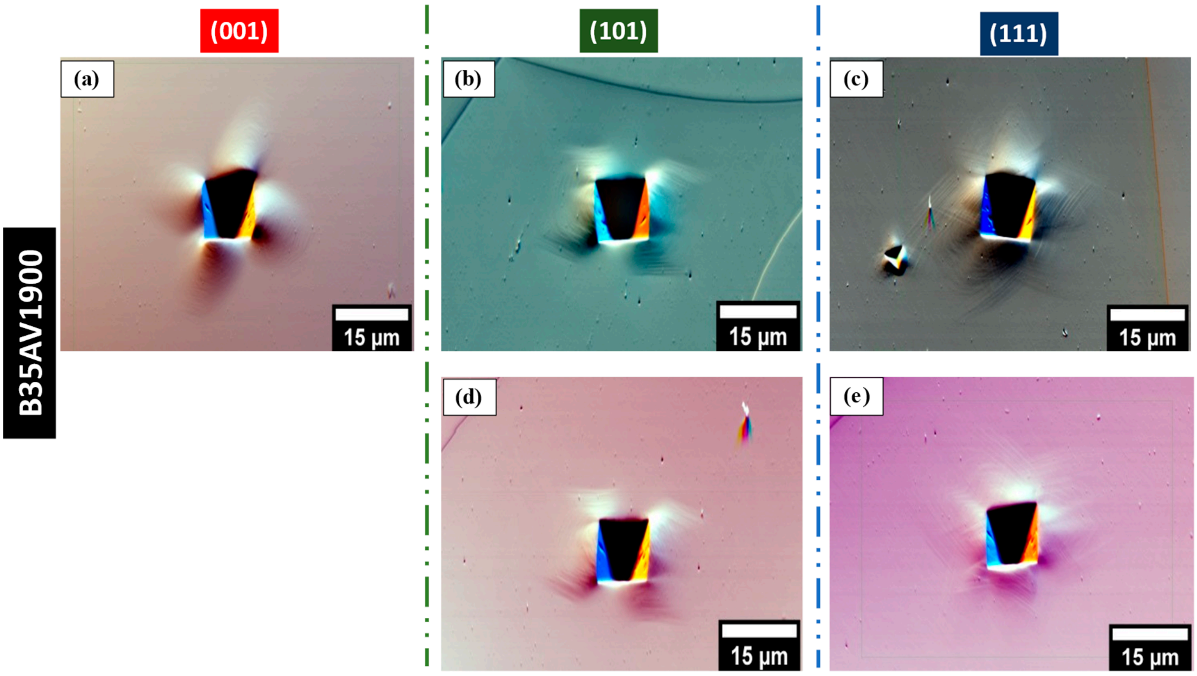

Figure S3 Grain-contrast (ARGUS forescatter) micrographs of representative Vickers micro-indentations in B35AV1900 grains selected near the (001), (101) and (111) crystallographic orientations. Panels (a-c) shows one representative indent for each orientation, while (d) and (e) show additional indents in grains selected near (101) and (111), respectively.

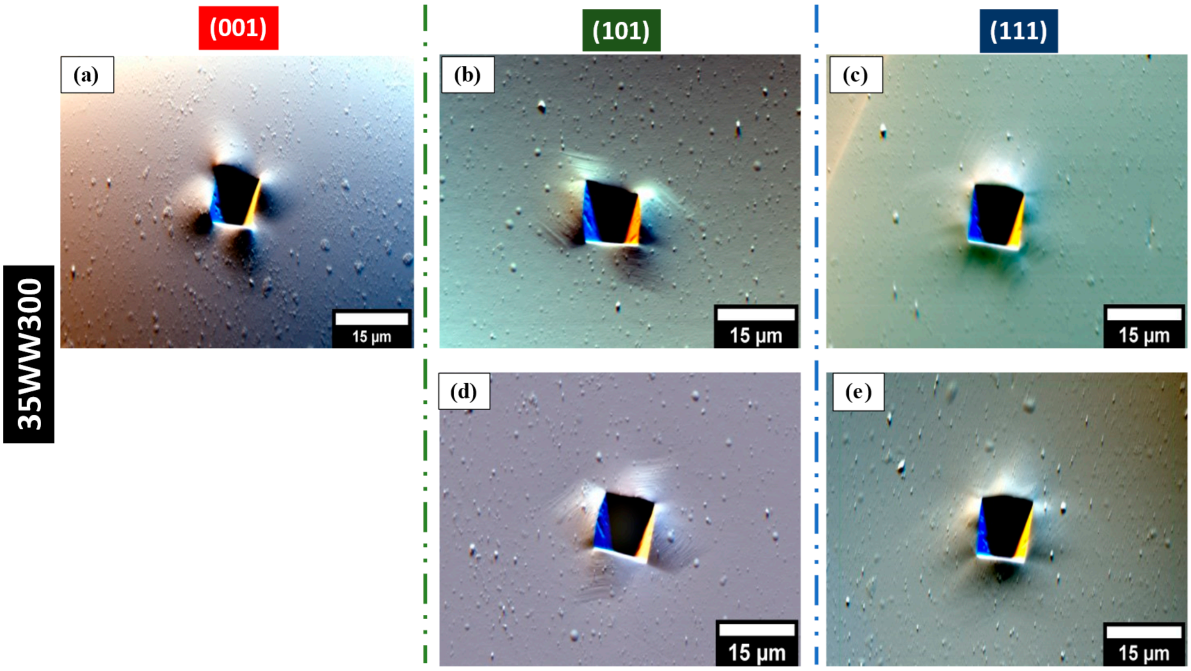

Figure S4 Grain-contrast (ARGUS forescatter) micrographs of representative Vickers micro-indentations in B35AV1900 grains selected near the (001), (101) and (111) crystallographic orientations. Panels (a-c) shows one representative indent for each orientation, while (d) and (e) show additional indents in grains selected near (101) and (111), respectively.

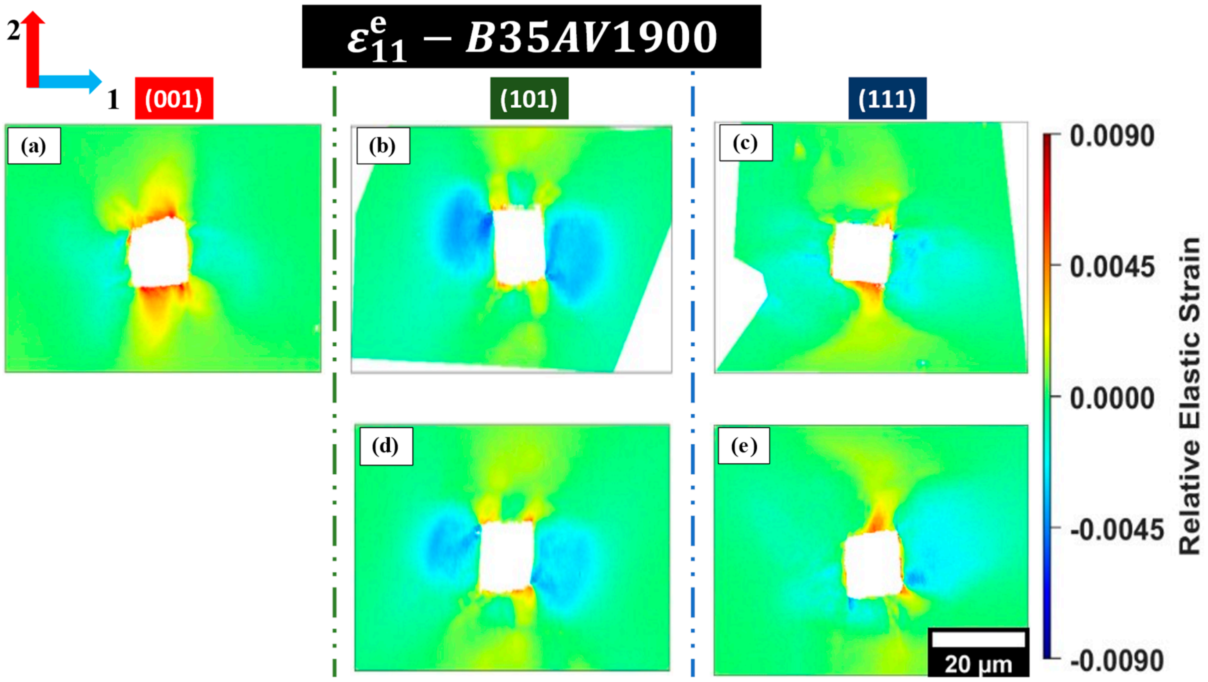

Figure S5 Additional ε^{e}_{11} residual in-plane elastic strain maps for extra indents in B35AV1900 grains oriented near (001), (101), and (111). One additional map is shown for near-(001), while two maps are shown for near-(101) and near-(111). All maps are plotted using a common colour scale to facilitate comparison of within-orientation variability and consistency of the observed strain-distribution trends. The 20 µm scale bar shown applies to panels.

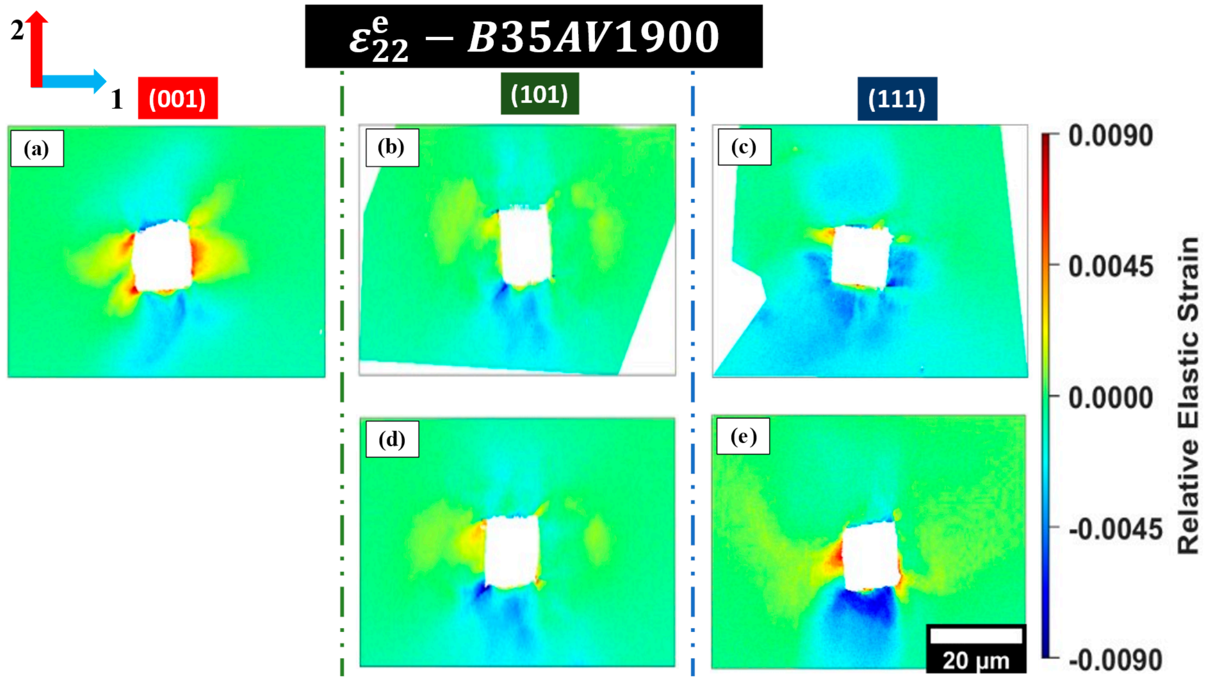

Figure S6 Additional ε^{e}_{22} residual in-plane elastic strain maps for extra indents in B35AV1900 grains oriented near (001), (101), and (111). One additional map is shown for near-(001), while two maps are shown for near-(101) and near-(111). All maps are plotted using a common colour scale to facilitate comparison of within-orientation variability and consistency of the observed strain-distribution trends. The 20 µm scale bar shown applies to panels.

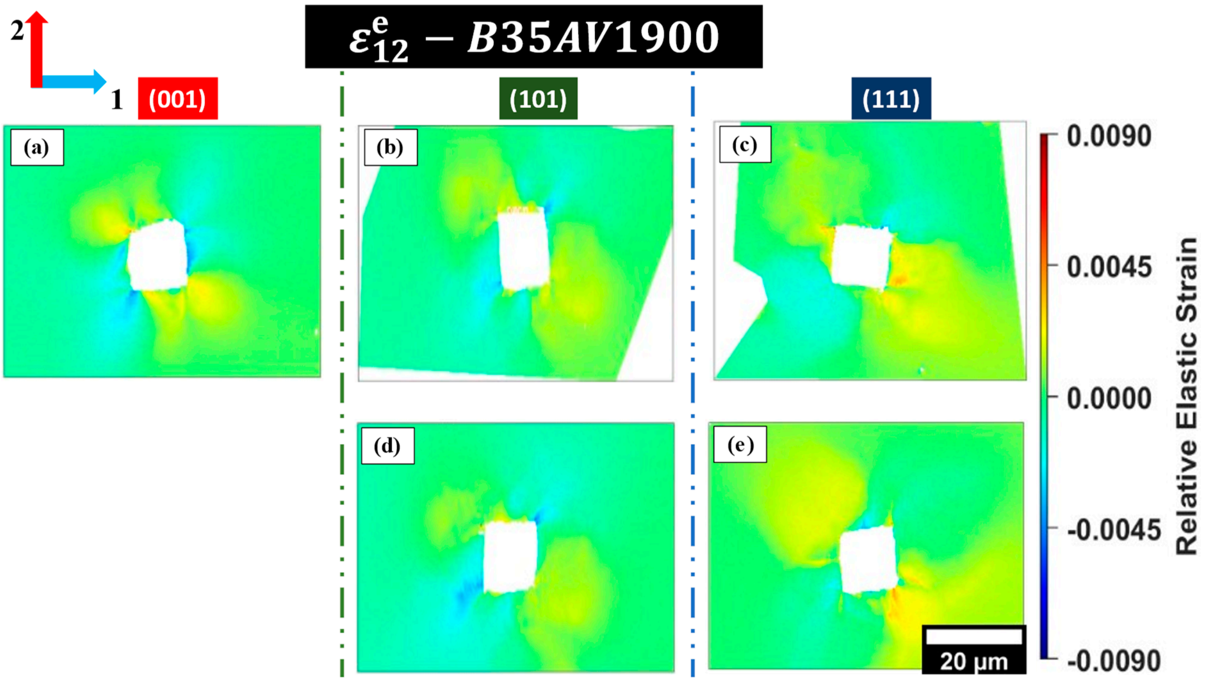

Figure S7 Additional ε^{e}_{12} residual in-plane elastic strain maps for extra indents in B35AV1900 grains oriented near (001), (101), and (111). One additional map is shown for near-(001), while two maps are shown for near-(101) and near-(111). All maps are plotted using a common colour scale to facilitate comparison of within-orientation variability and consistency of the observed strain-distribution trends. The 20 µm scale bar shown applies to panels.

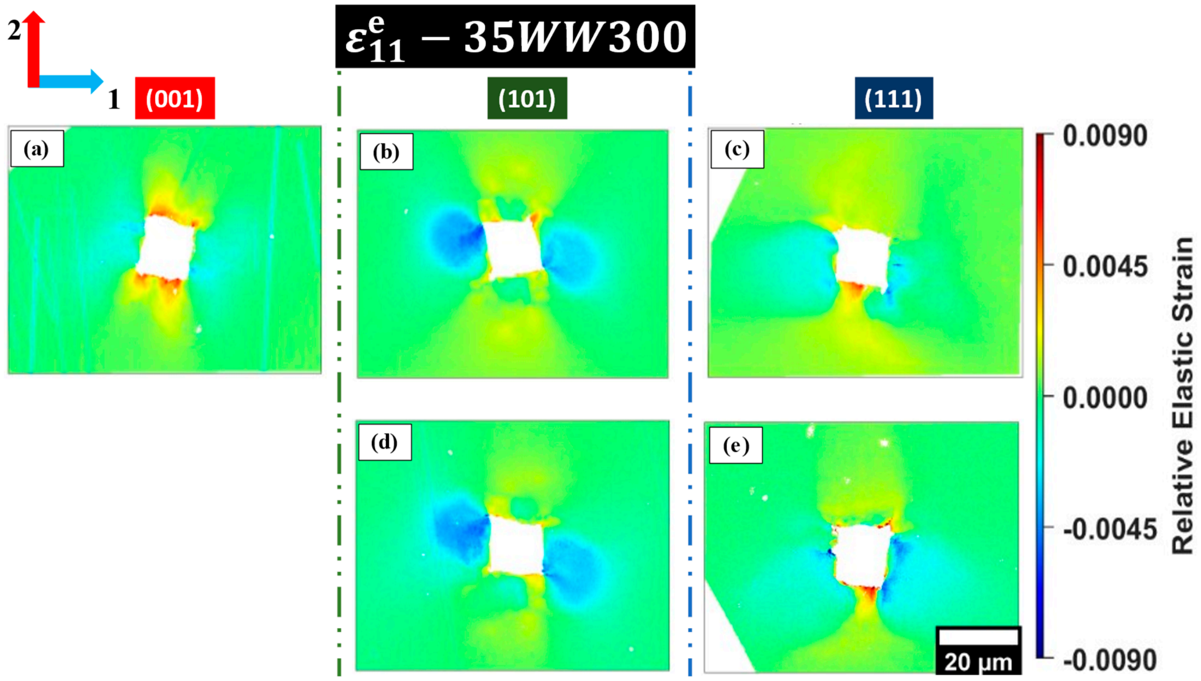

Figure S8 Additional ε_{11}^{e} residual in-plane elastic strain maps for extra indents in 35WW300 grains oriented near (001), (101), and (111). One additional map is shown for near-(001), while two maps are shown for near-(101) and near-(111). All maps are plotted using a common colour scale to facilitate comparison of within-orientation variability and consistency of the observed strain-distribution trends. The 20 μm scale bar shown applies to panels.

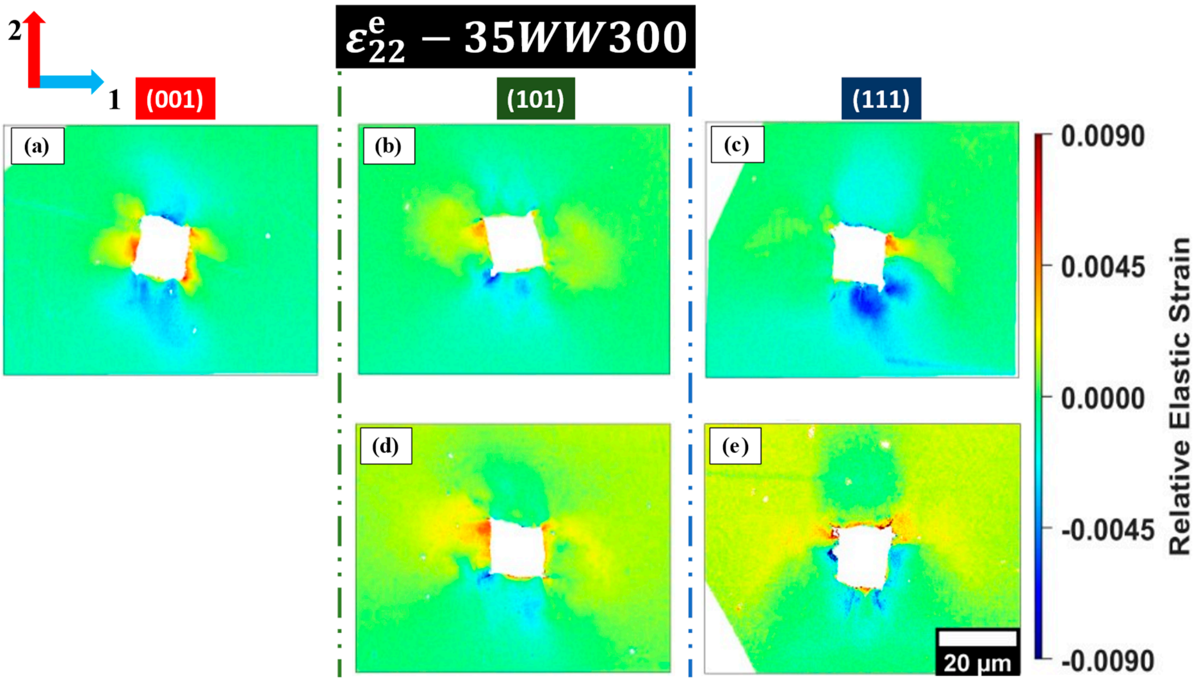

Figure S9 Additional ε_{22}^{e} residual in-plane elastic strain maps for extra indents in 35WW300 grains oriented near (001), (101), and (111). One additional map is shown for near-(001), while two maps are shown for near-(101) and

near-(111). All maps are plotted using a common colour scale to facilitate comparison of within-orientation variability and consistency of the observed strain-distribution trends. The 20 μm scale bar shown applies to panels.

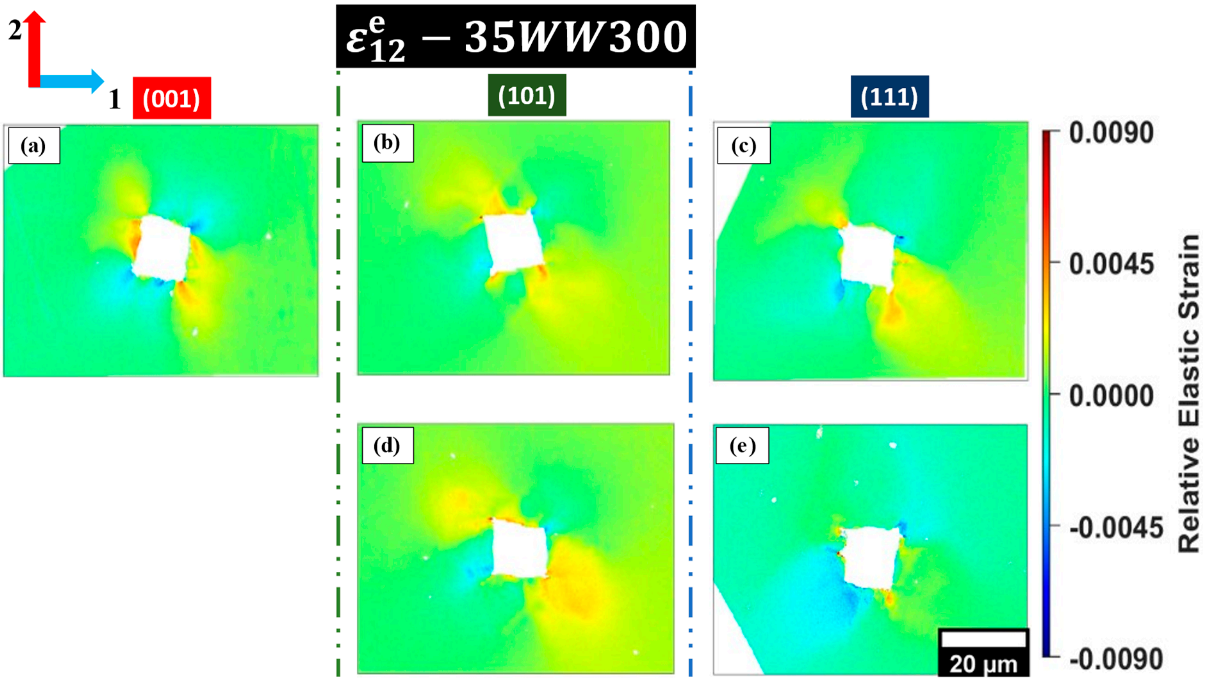

Figure S10 Additional ε^{e}_{12} residual in-plane elastic strain maps for extra indents in 35WW300 grains oriented near (001), (101), and (111). One additional map is shown for near-(001), while two maps are shown for near-(101) and near-(111). All maps are plotted using a common colour scale to facilitate comparison of within-orientation variability and consistency of the observed strain-distribution trends. The 20 μm scale bar shown applies to panels.

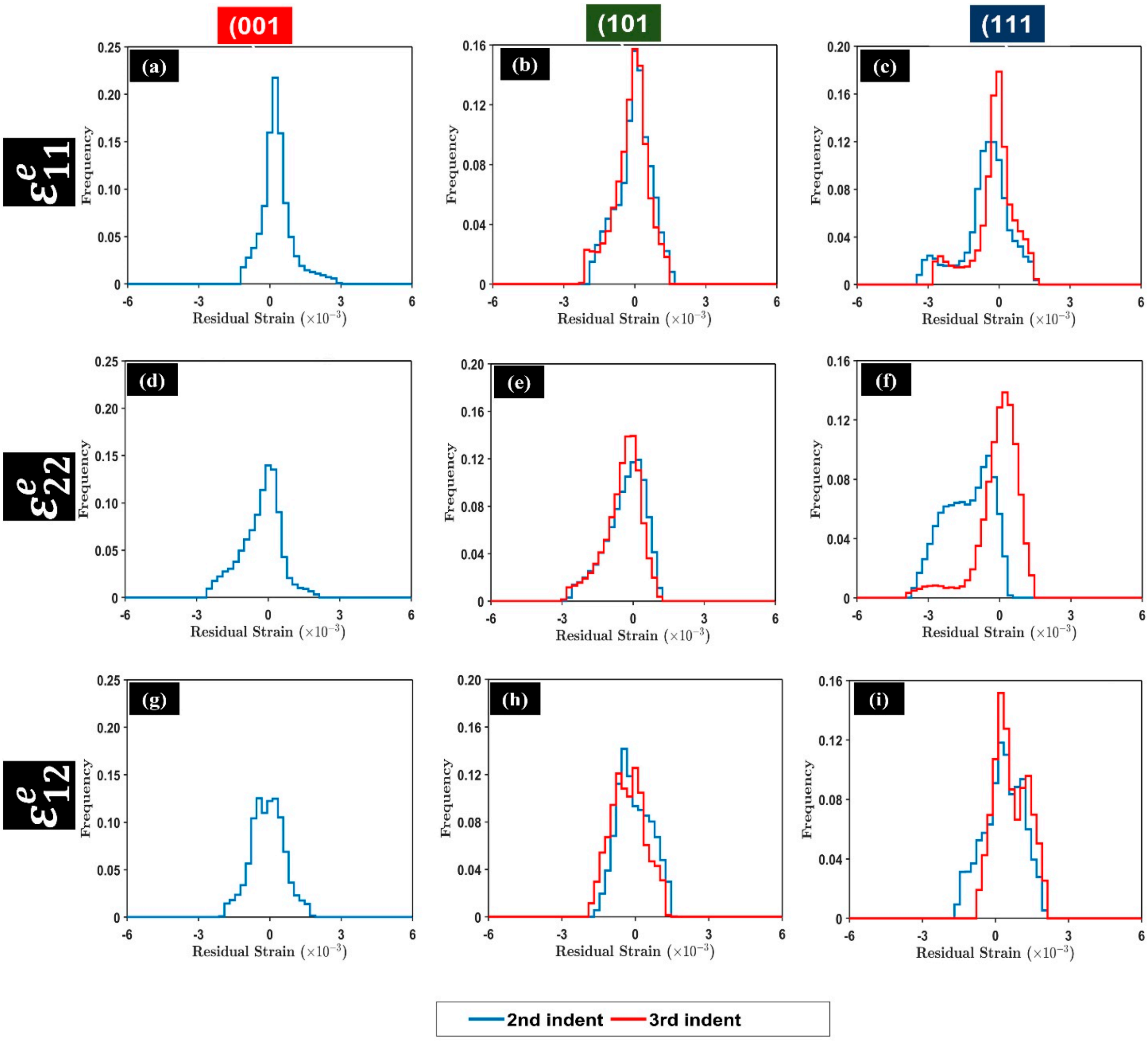

Figure S11 Frequency distributions of the residual in-plane elastic strain components ε_{11}^{e} , ε_{22}^{e} and ε_{12}^{e} are presented for additional indents in B35AV1900 grains oriented near (001), (101) and (111). For the near-(001) grain, one additional indent is displayed. For the near-(101) and near-(111) grains, the blue and red curves represent to the second and third indents, respectively. These plots demonstrate the variability within each orientation and the consistency of strain-distribution trends across repeated indents.

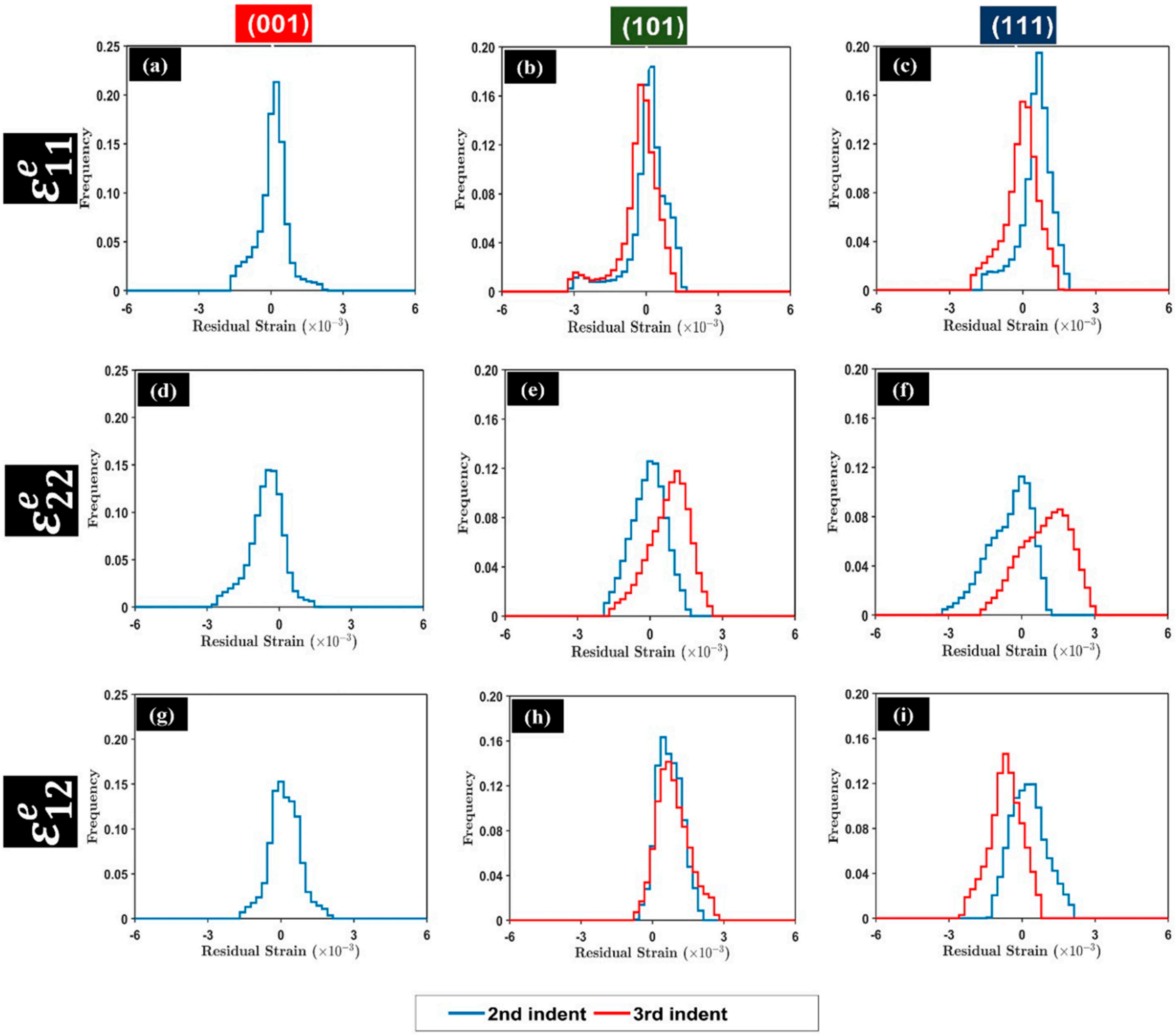

Figure S12 Frequency distributions of the residual in-plane elastic strain components ε_{11}^{e} , ε_{22}^{e} and ε_{12}^{e} are presented for additional indents in 35WW300 grains oriented near (001), (101) and (111). For the near-(001) grain, one additional indent is displayed. For the near-(101) and near-(111) grains, the blue and red curves represent to the second and third indents, respectively. These plots demonstrate the variability within each orientation and the consistency of strain-distribution trends across repeated indents.

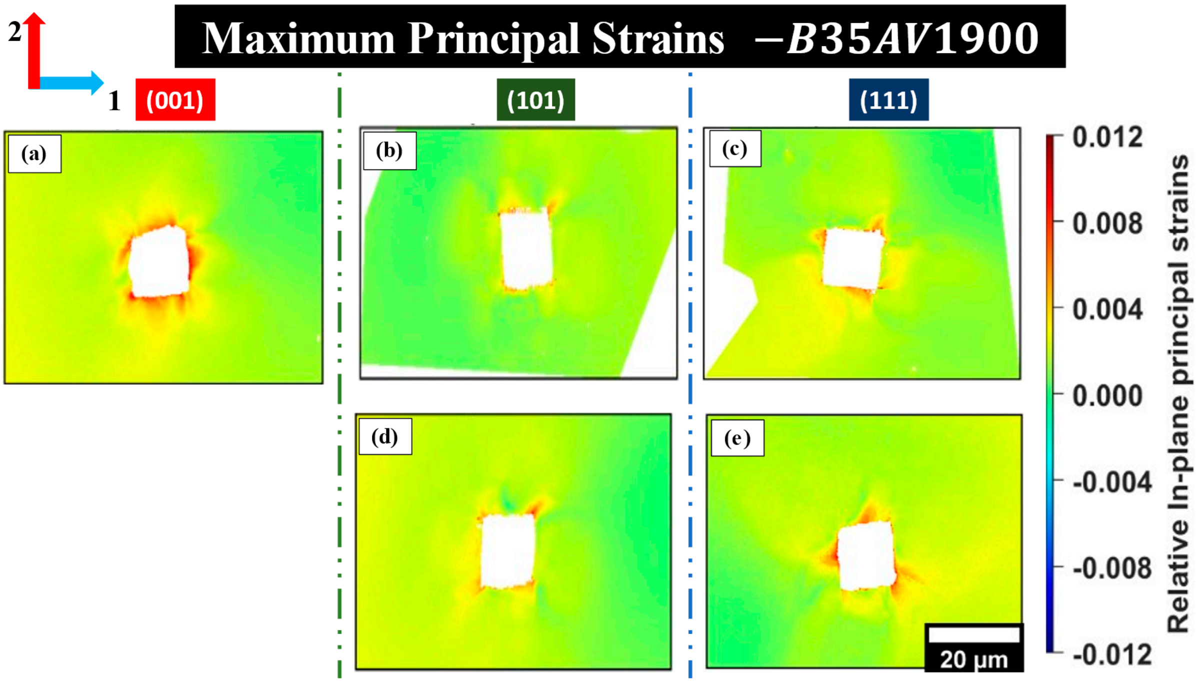

Figure S13 Additional in-plane relative maximum principal strain maps are provided for extra indents in B35AV1900 grains oriented near (001), (101) and (111). One additional map is shown for near-(001), while two maps are shown for near-(101) and near-(111). All maps are plotted using a common colour scale to facilitate comparison of within-orientation variability and consistency in strain-distribution trends. The 20 µm scale bar applies to all panels.

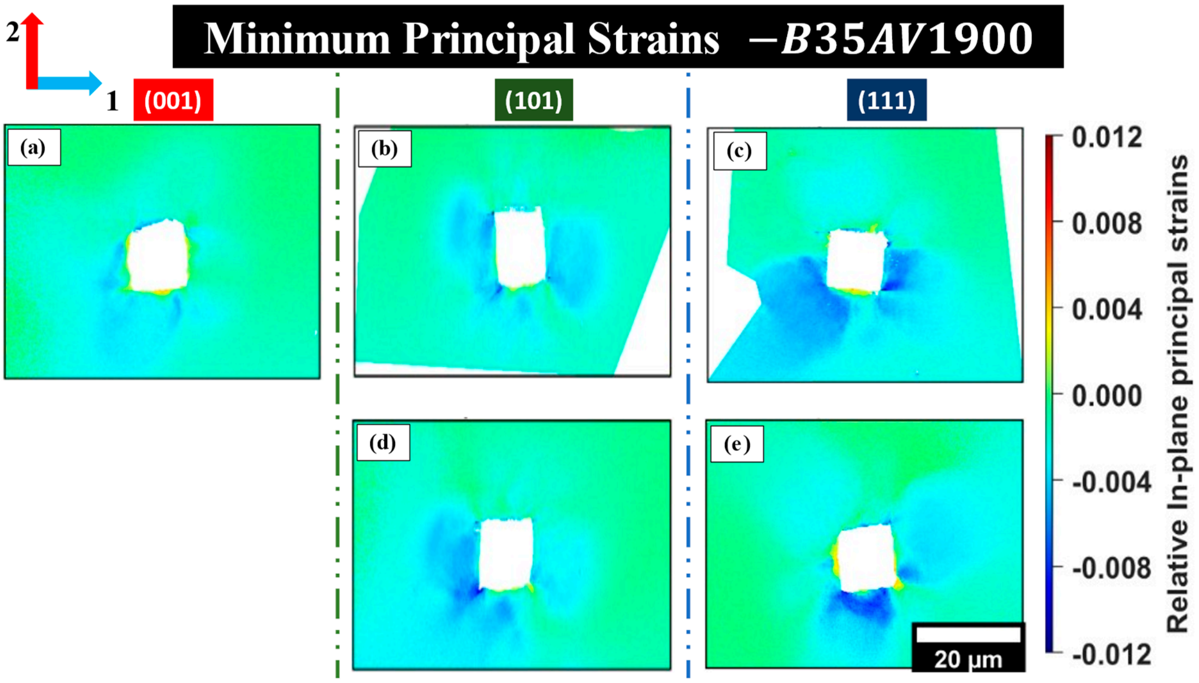

Figure S14 Additional in-plane relative minimum principal strain maps are provided for extra indents in B35AV1900 grains oriented near (001), (101) and (111). One additional map is shown for near-(001), while two maps are shown for near-(101) and near-(111). All maps are plotted using a common colour scale to facilitate comparison of within-orientation variability and consistency in strain-distribution trends. The 20 µm scale bar applies to all panels.

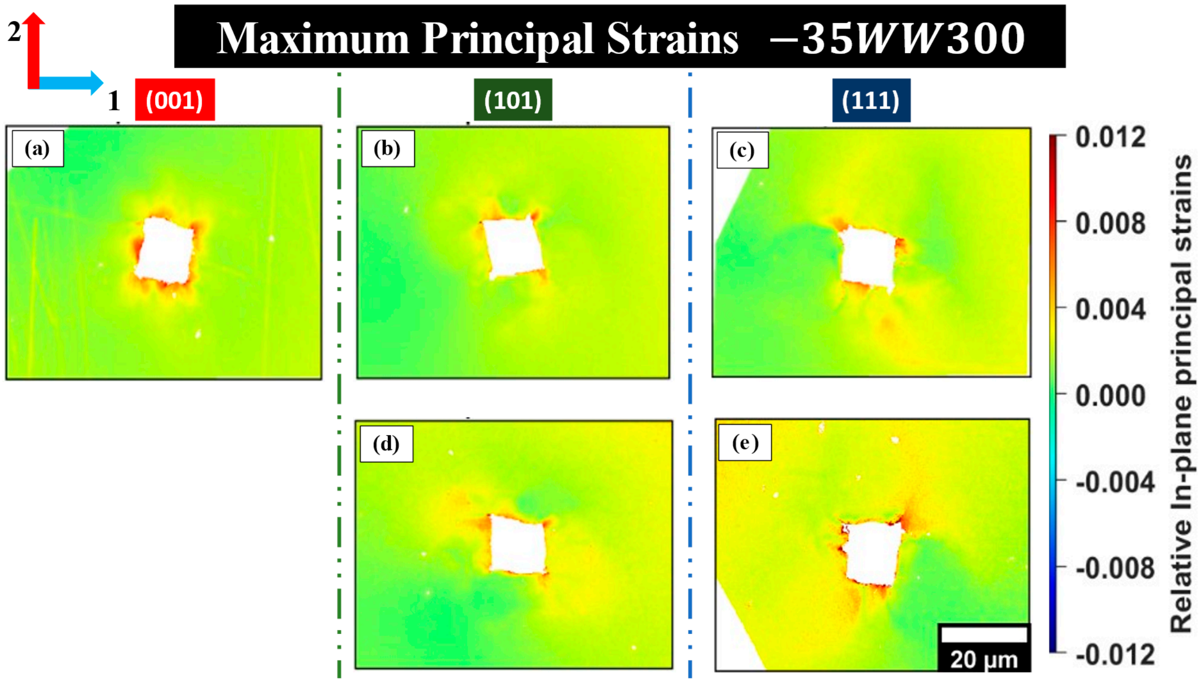

Figure S15 Additional in-plane relative maximum principal strain maps are provided for extra indents in 35WW300 grains oriented near (001), (101) and (111). One additional map is shown for near-(001), while two maps are shown for near-(101) and near-(111). All maps are plotted using a common colour scale to facilitate comparison of within-orientation variability and consistency in strain-distribution trends. The 20 μm scale bar applies to all panels.

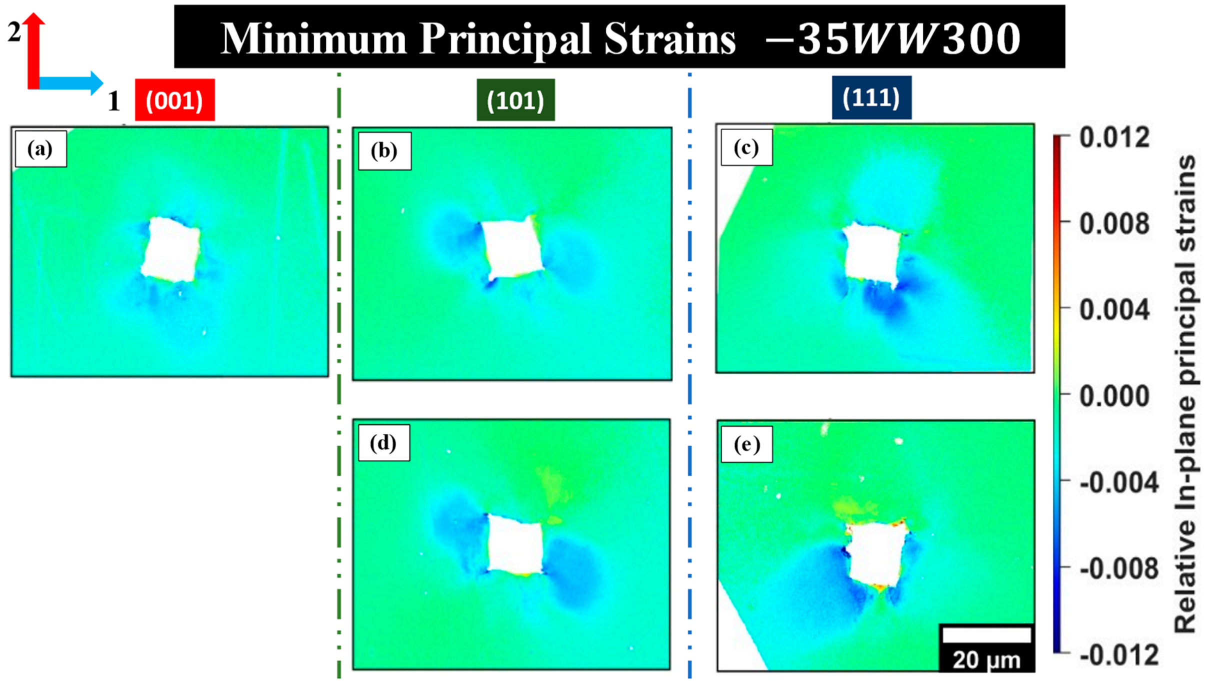

Figure S16 Additional in-plane relative minimum principal strain maps are provided for extra indents in 35WW300 grains oriented near (001), (101) and (111). One additional map is shown for near-(001), while two maps are shown for near-(101) and near-(111). All maps are plotted using a common colour scale to facilitate comparison of within-orientation variability and consistency in strain-distribution trends. The 20 μm scale bar applies to all panels.
